# Role of mesenchymal stem cell-derived soluble factors and folic acid in wound healing

**DOI:** 10.3906/sag-1901-231

**Published:** 2019-06-18

**Authors:** Ahu PAKDEMİRLİ, Feriha TOKSÖZ, Aslıhan KARADAĞ, Hüseyin Koray MISIRLIOĞLU, Yasemin BAŞBINAR, Hülya ELLİDOKUZ, Osman AÇIKGÖZ

**Affiliations:** 1 Department of First and Emergency Aid, Vocational School of Health Services, Dokuz Eylül University, İzmir Turkey; 2 Tailor of Science Biotechnology Innovation Inc., Dokuz Eylül Technology Development Zone-DEPARK Inc., Dokuz Eylül University, İzmir Turkey; 3 Department of Translational Oncology, Institute of Oncology, Dokuz Eylül University, İzmir Turkey; 4 Personalized Medicine and Pharmacogenomics Research Center, Dokuz Eylül University, İzmir Turkey; 5 Department of Preventive Oncology, Institute of Oncology, School of Medicine, Dokuz Eylül University, İzmir Turkey; 6 Department of Physiology, Faculty of Medicine, Dokuz Eylül University, İzmir Turkey

**Keywords:** Mesenchymal stem cell, mesenchymal stem cell-derived soluble factor, wound healing, folic acid, cell differentiation

## Abstract

**Background/aim:**

Mesenchymal stem cells (MSCs) are a type of adult stem cell consisting of a heterogeneous subset of stromal stem cells that can be isolated from adult tissues. Folic acid is another important contributor to tissue regeneration and repair, which affects the synthesis of some building block molecules used for wound healing. In this study, we examine the effect of folic acid and MSC-derived soluble factors in the wound healing model.

**Materials and methods:**

Human umbilical vein endothelial cells (HUVECs) and bone marrow-derived MSCs (BMSCs) were cultured for this study. Cell proliferation analysis was done with xCELLigence RTCA. After 48 h of cultivation, the cell culture medium was collected as MSC conditional medium containing mesenchymal stem cell-derived soluble factors (MDFs). Different concentrations of MDFs (12%, 25%, 50%, 75%, and 100%) were applied to the HUVEC cell line. Folic acid (25, 30, 50, 60, 75, 90, and 100 µM) was tested by application of three different groups (control, 25 µM folic acid, 625 µM folic acid inhibitors) for proliferation on the HUVEC cell line. The combined effects of folic acid and MDFs were tested on the HUVEC cell line with 25 µM folic acid and 50 µM MDFs. All data were statistically analyzed using SPSS 15.0 for Windows.

**Results:**

Significant differences were observed between controls and cells treated with folic acid, as well as between controls and both folic acid and MDFs (P < 0.05). Among the treated groups, the fastest wound closure rate was seen in cells treated with both folic acid and MDFs.

**Conclusion:**

The results show that both folic acid and MDFs increased the wound healing rate in HUVECs when they were used separately. The strongest benefits were seen in treatment using folic acid and MDFs together.

## 1. Introduction

Wound healing is an important and complicated process that involves cell proliferation, migration, angiogenesis, reepithelization, and tissue remodeling as a result of complex interactions between growth factors, extracellular matrix molecules, and cells [1]. Stem cells have emerged as a promising treatment for wounds due to their ability to renew and differentiate themselves. Adult stem cells such as bone marrow-derived mesenchymal stem cells (BMSCs) and adipose-derived mesenchymal stem cells (ASCs) are not hard to obtain due to ethical concerns and availability like embryonic stem cells. BMSCs and ASCs can be easily isolated in adults [2,3].

Mesenchymal stem cells (MSCs), a heterogeneous subset of stromal stem cells, are multipotent adult stem cells that are present in multiple tissues, including the umbilical cord, bone marrow, and fat tissue. They are reproducible and have a high potential for use in different areas [4]. Because of their stromal origin, they are supportive and durable. 

MSCs have the potential to differentiate into different cell types primarily in connective tissue when provided with appropriate microenvironmental conditions [5]. They have osteogenic, adipogenic, chondrogenic, and myogenic differentiation capacities in in vitro conditions with appropriate stimuli. MSCs can also transform into pancreatic beta cells, hepatocytes, endothelium, and epithelioid cells [6]. MSC differentiation is a process that involves the release of mesenchymal stem cell-derived soluble factors (MDFs). MDFs are rich in materials that are key substances in wound healing, including growth factors such as transforming growth factor (TGF-β), platelet-derived growth factor (PDGF), keratinocyte growth factor (KGF), vascular endothelial growth factor (VEGF), epidermal growth factor (EGF), fibroblast growth factor (FGF), connective tissue growth factor (CTGF), insulin-like growth factor (IGF), and colony-stimulating factors (CSFs); cytokines such as tumor necrosis factor α (TNF-α), interleukin-1, interleukin-6, interleukin-8, and the angioinflammatory interleukins (interleukin-4 and interleukin-10); and interferons [7].

MSCs interact with the innate and adaptive immune system, causing the modulation of many effector functions. Whenever tissue damage occurs, these stem cells move to the area and start to repair the damage [8,9]. They prevent the release of proinflammatory cytokines and induce peripheral tolerance to promote the survival of damaged cells [10]. Preclinical and clinical studies suggest that transplanted MSCs alter the phenotype. How immune cells function is largely due to the production of MDFs. The expression varies depending on the pathologic conditions to which MSCs are exposed [11].

Folic acid is a water-soluble vitamin in the B complex that requires exogenous uptake for health, growth, and development. It is required as the precursor of cofactors for single-carbon donors in the synthesis of DNA bases and some other essential biomolecules [12]. It is involved in many metabolic functions, including DNA replication and repair, methylation, and synthesis of nucleotides and some amino acids.

Folic acid, which is one of the important factors in tissue regeneration and repair, is being investigated due to its role in the synthesis of both building-block molecules and their effects on differentiation [13]. Folic acid induces the differentiation of neural stem cells into neurons via DNA methyltransferases and plays an important role in stem cell proliferation and differentiation [14]. In this study, the capacity of MSCs to secrete endothelial damage by folic acid as a result of coculture was investigated in an in vitro wound healing model.

## 2. Materials and methods

### 2.1. Cell culture

Human umbilical vein endothelial cells (HUVECs) (ATCC CRL-1730) were obtained from the American Type Culture Collection (Rockville, MD, USA) and cultured in low-glucose Dulbecco’s Modified Eagle’s Medium (DMEM) containing 10% fetal bovine serum (FBS, Cegrogen Biotech GmbH, Stadtallendorf, Germany) and 1% penicillin/streptomycin (Biochrom GmbH, Berlin, Germany). Cells were incubated at 37 °C in 5% CO2 in humidified air.

BMSCs were cultured in Mesenchymal Stem Cell Basal Medium for Adipose, Umbilical and Bone Marrow-derived MSCs (ATCC PCS-500-030), containing 10% FBS (Biochrom GmbH), 1% penicillin/streptomycin (Biochrom GmbH), and L-glutamine (4.5 mM) (Biochrom GmbH). Cells were incubated at 37 °C in 5% CO2 in humidified air.

### 2.2. Cell proliferation and wound healing assay

The cells were incubated in the appropriate medium for cell proliferation analysis with the xCELLigence RTCA real-time cell analyzer. HUVECs were seeded at 5000 cells per well in 96-well culture plates. Cell index-cell content (CI) values were recorded every 15 min for 48 h. The wells containing DMEM were used as negative controls for resistivity baseline measurements.

After cell proliferation analysis, cells were seeded at 105 cells per well in 6-well culture plates. When they became confluent, a wound was created with a 200 µL pipette. Different concentrations of folic acid (25, 30, 50, 60, 75, 90, 100 µM) were added into the wells. Cells were incubated at 37 °C and 5% CO2 in humidified air. Images of wound closure were taken at 0, 24, and 48 h using the JuLI Br live cell movie analyzer (NanoEnTek, Korea) with barrels attached. The amount of wound closure was calculated with ImageJ software 1.49.

### 2.3. Coculture modeling and MDF production protocols

The HUVEC cell line was plated on Matrigel in a 2-well culture well. L-DMEM medium was added to the upper chamber. The BMSC cell line was seeded in the bottom chamber at 37 °C in 5% CO2 for 48 h. As a test variable, folic acid was applied to the bottom chamber. After 48 h, the cell-culture medium was collected as MSC culture medium) containing MDFs and stored at –80 °C. Different concentrations of this medium (12%, 25%, 50%, 75%, and 100%) were tested for proliferation in the HUVEC cell line. Wound healing and combined effects were based on EC50 dose. After another 48 h, the stem cells in the lower chamber were observed under an inverted microscope.

### 2.4. Statistical analysis

SPSS 15.0 (SPSS Inc., Chicago, IL, USA) was used for the data analysis. The mean ± standard error (SE) was used for numerical values found in the figures and text. A nonparametric Mann–Whitney U test was used to determine the statistical significance. P < 0.05 was considered statistically signiﬁcant.

## 3. Results

### 3.1. HUVEC cell line proliferation assays

**Figure 1:**
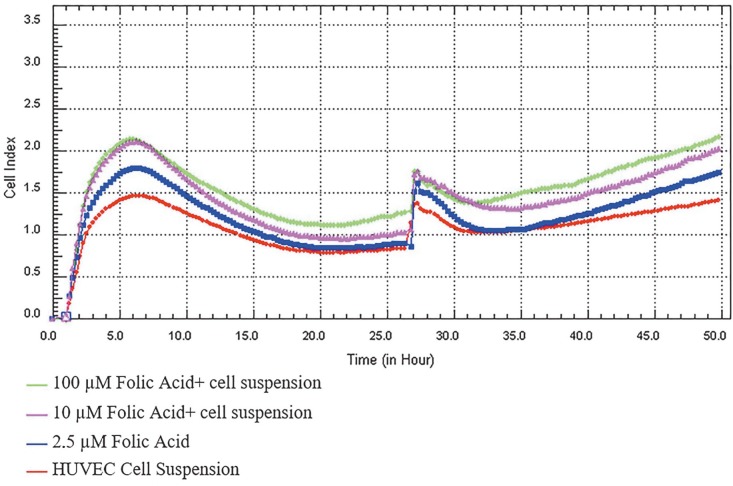
Folic acid proliferation analysis. Cell proliferations were measured in real time for 48 h in the studied concentration range of folic acid (2.5–100 μM). Cell index (CI), which is the change in the electrical impedance of the cell increase, is graphed against time. When the trend change between recurrent measurements was analyzed using Mauchly’s sphericity test, the 100 μM folate group was found to be significantly higher than the control group (P < 0.05).

Folic acid increased cell proliferation in the HUVEC cell line. In this study, according to the analysis of the results by the xCELLigence RTCA device, the cell index was used to determine the EC50 dose as 50 µL (25% concentration) as shown in Figure 1.

**Figure 2:**
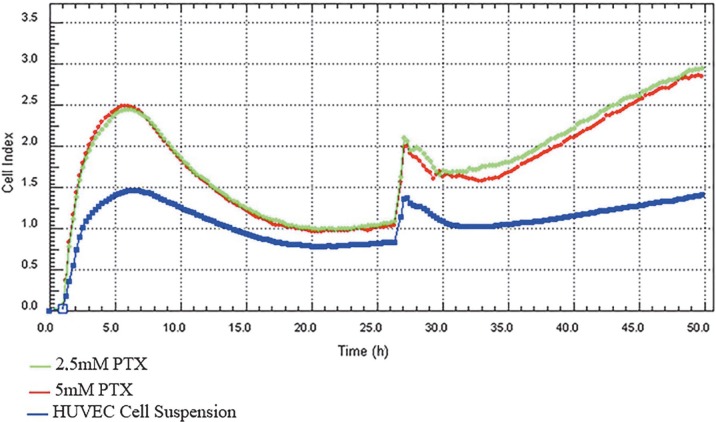
Pemetrexed (folic acid metabolism inhibitor) effect. PTX (0.25–20 μM, low dose effect; 125 μM–2.5 mM, high dose effect) cell proliferations were measured in real time for 7 h. A nontoxic dose was selected for HUVECs for combined effect. When the trend change between recurrent measurements was analyzed using Mauchly’s sphericity test, the 5 and 2.5 mM PTX groups were significantly higher than the control group (P < 0.05).

In order to determine the nontoxic doses of folic acid inhibitors in the HUVEC cell line, proliferation studies were performed with low and high doses according to the literature [15]. Experiments were performed at 7 different concentrations in each dose range. The highest nontoxic HUVEC cell line for both inhibitors was 5 mM as shown in Figure 2.

The proliferative effect of folic acid is blocked in the HUVEC cell line by the combination of PMX and MTX, an antagonist and an inhibitor of folic acid, respectively.

**Figure 3:**
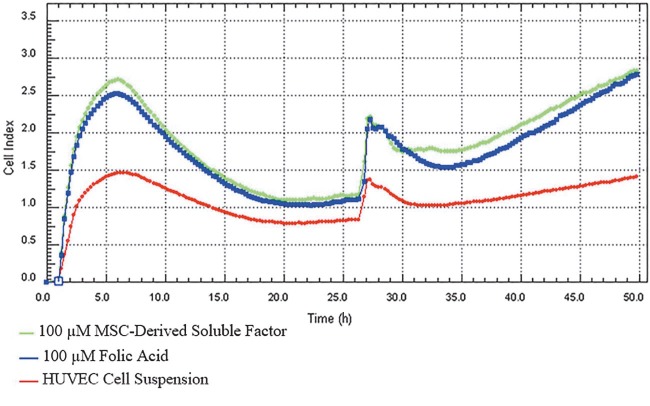
Folic acid + MSC-derived soluble factor effect. Folic acid (2.5–100 μM) was added to the bottom chamber in the coculture medium + 50 μL mesenchymal stem cell culture medium at each concentration; cell proliferation was measured in real time for 48 h at 7 concentrations. The trend change between repeating measurements was analyzed using Mauchly’s sphericity test (P < 0.05).

The proliferative effect was potentiated by the mediators released into the coculture medium as defined in the MDF production protocol and increased proliferation. Figure 3 shows the time-dependent effect of cell proliferation. 

### 3.2. Wound healing

#### 3.2.1. Folic acid effect

In the in vitro endothelial wound model of the HUVEC cell line, the effects of folic acid were investigated, which is a factor of the effects of MSCs, folic acid, and MDFs on the environment.

**Figure 4:**
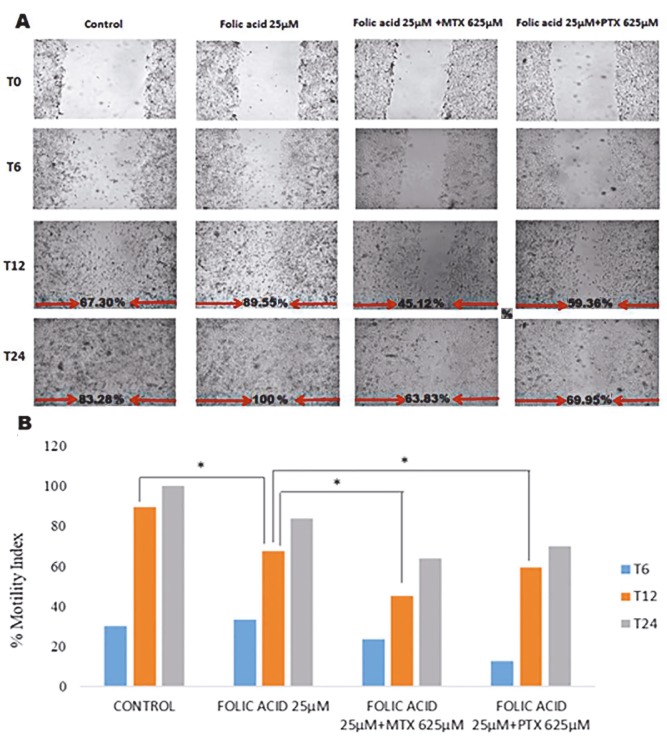
Folic acid effect in the HUVEC wound healing model. The effect of folic acid with PTX and MTX on cells was monitored for 24 h. A) Real-time images of wound healing; B) wound closure rates are given as percentage motility index. t: Folic acid group against control; *: MTX against folic acid group; &: PTX against folic acid group.

In the wound healing model, an EC50 dose of folic acid was used. Wound healing was measured at 0, 6, 12, and 24 h as shown in Figure 4. The wound healing rate was calculated as percentage motility index. After 24 h, the motility index was 73.64% in the cell culture medium as a control, 100% in the folic acid group, 63.82% in the folic acid combined with MTX group, and 69.95% in the folic acid combined with PTX group.

#### 3.2.2. Mesenchymal stem cell-derived soluble factor effect

**Figure 5:**
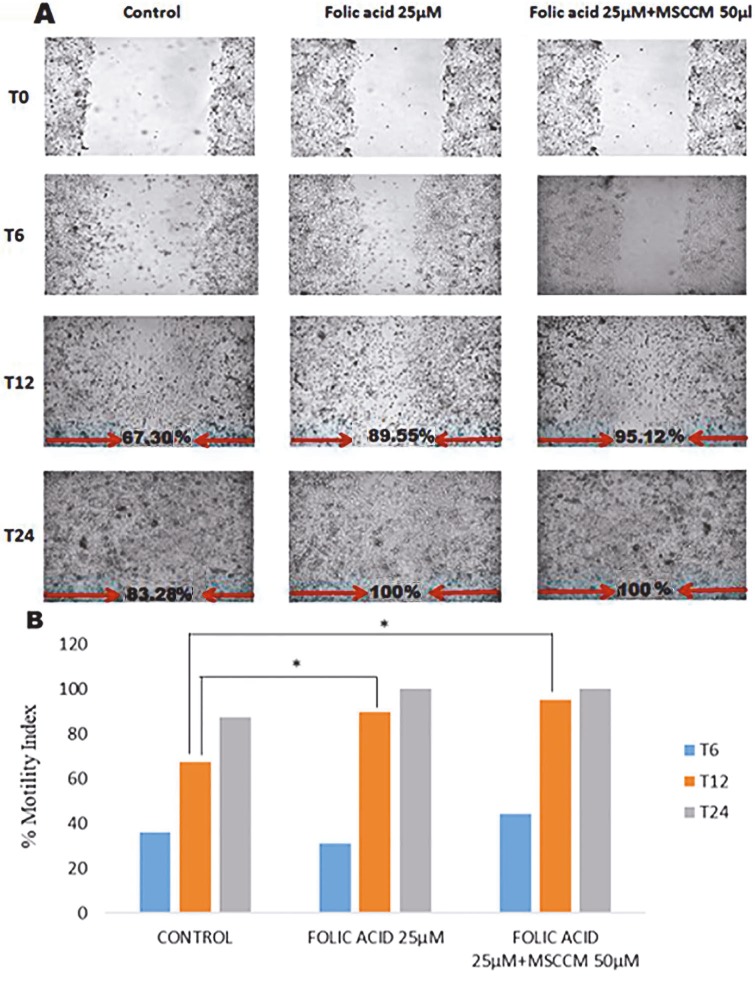
The effect of mesenchymal stem cells in the HUVEC wound healing model. MDFs, folic acid, and folic acid with MDFs in the coculture model wound healing effects were monitored for 24 h. A) Real-time images of wound healing; B) wound closure rates. t: Folic acid group against MDFs; *: folate + MDFs group against MDFs.

In the wound healing model, a 25 µM dose of folic acid, which increased the cell proliferation by 50%, and MDFs (50 µL) obtained from MSC conditional medium and 25 µM folic acid-added MDFs (50 µL) in the coculture model were selected. Wound healing was observed at 0, 6, 12, and 24 h. Wound healing rate was calculated as percentage motility index. As shown in Figure 5, after 24 h, wound healing with MDFs in the coculture model was calculated as 83.74%, It was 100% in the folic acid group and 100% in the group with folic acid combined with MSCs. After 12 h, wound healing was 67.30%, 89.56%, and 95.12%, respectively.

Wound healing rate increases with the addition of MDFs. In the positive control group with high FBS (20%), the closure rate of 73.64% after 24 h increased to 83.74% with the effect of MSCs. MDFs obtained by the addition of folic acid to the coculture medium further increased the wound healing rate in response to the folic acid.

## 4. Discussion

This study investigated the role of folic acid on regeneration in vascular tissue and the healing ability of different soluble factors associated with MSCs on wound healing. The results show that MDFs have an enhancing effect on cell proliferation and migration. Folic acid also increases the proliferation of vascular endothelial cells and induces the repair of vascular damage.

The presence of PMX and MTX, an antagonist and an inhibitor of folic acid, limit the proliferation and the wound healing. These data also support that folic acid has a role in the regeneration.

According to the results of this study, the effect of folic acid on the proliferation and wound healing in vascular endothelial cells decreases in the presence of folic acid antagonists and inhibitors. This indicates that folic acid is effective in improving endothelial damage.

There is a considerable impact on epidermal cells’ migration and mitotic division rates during wound healing. It was suggested that epidermal repair in wound healing is affected by the movement of epidermal cells over each other upon the surface of the wound [16]. Folic acid is also known to be essential for DNA synthesis and repair [17]. The folic acid may be reduced to active tetrahydrofolic acid in response to folic acid reductase in vivo. Since tetrahydrofolic acid is the main carrier of a carbon unit and plays a role in the synthesis of purine and pyrimidine and in the mutual transformation of amino acids, lack of this vitamin in vivo may impair the transmission of a carbon unit and affect the nucleic acid synthesis and amino acid metabolism necessary for cell proliferation, tissue growth, and development. Therefore, folic acid plays a critical role in the development of proliferation and differentiation of stem cells [14]. The presence of folic acid in cell division during wound healing increased the rate of healing due to its increasing effect on cell division.

Regeneration procedures in wound healing normally occur through the extracellular matrix and are caused by complex interactions between cells and paracrine factors. When the effect of paracrine factors secreted by BM-MSCs on wound healing was examined by Chen et al. [18], it was observed that the cells had healing effects. The coculture model and MSC-derived secretory factors have been shown to increase the effect of wound healing on cell migration by the paracrine effect. The content of MDFs will be important in this context.

In addition, it was determined that the combined application was more effective than stem cell injection and folic acid in individual applications. Heseker noted that supplementation with folic acid significantly reduces the risk of neural tube defects and helps with healing. The results of this study confirm this research [19].

This study’s results are proven to be accurate for the in vitro wound healing model. It serves as a contribution to the literature on in vivo (phase 0) studies.

## Acknowledgment

This work was supported by Dokuz Eylül University Scientific Research Coordination Unit (Project Number: 2017.KB.SAG.003).
